# Health technology management: the experience of a managed access approach to the reimbursement of dupilumab in Ireland

**DOI:** 10.1007/s11845-023-03378-7

**Published:** 2023-05-09

**Authors:** Rosealeen Barrett, Michael Barry, Laura McCullagh

**Affiliations:** 1 Department of Pharmacology and Therapeutics, Trinity College Dublin, Trinity Centre for Health Sciences, St. James’s Hospital, Dublin 8, Ireland; 2 Medicines Management Programme, Health Service Executive, St. James’s Hospital, Dublin 8, Ireland; 3 National Centre for Pharmacoeconomics, St. James’s Hospital, Dublin 8, Ireland

**Keywords:** Atopic dermatitis, Cost-effective prescribing, Drug optimisation, Evidence-based prescribing, Health Technology Management, Medicines Management

## Abstract

**Background:**

Dupilumab was the first biological medicine to receive marketing authorisation, in Ireland, for the treatment of atopic dermatitis (AD). In 2019, Ireland’s National Centre for Pharmacoeconomics recommended that dupilumab not be reimbursed at the submitted price; it was not cost effective. Following confidential price negotiations, the Health Service Executive (HSE) reimbursed dupilumab subject to a HSE-Managed Access Protocol (MAP). Patients with refractory, moderate-to-severe AD were deemed eligible to receive treatment under the MAP; the cohort for which dupilumab is expected to be most effective and most cost effective (versus standard of care). Approval, for treatment, is granted on an individual patient basis by the HSE-Medicines Management Programme.

**Aims:**

Applications for approval of treatment with dupilumab were analysed to determine the percentage of patients deemed eligible. Key characteristics of this population were investigated.

**Methods:**

Data from individual patient applications were analysed. Key characteristics of the approved population were investigated using IBM SPSS Statistics^®^ version 27.

Data were derived from the HSE-Primary Care Reimbursement Service pharmacy claims database. The number of patients who received dupilumab over the study period was determined.

**Results:**

In total, 96% of submitted applications were deemed eligible. Of these, 65% were male and 87% were adults. In the main, the approved patient population had severe refractory AD; the mean Eczema Area Severity Index score was 28.72.

**Conclusion:**

The majority of applications submitted were approved. This work highlights how a MAP can facilitate access to treatment in patients who are deemed eligible whilst containing overall expenditure.

## Introduction

### Atopic dermatitis

Atopic dermatitis (AD) is an inflammatory, pruritic and chronic or chronically relapsing skin disease. AD is one of the most common non-communicable skin diseases; as a heterogeneous and intermittent condition, reported incidence rates vary. European prevalence rates are approximately 20% in children and up to 8% in adults [1]. Systemic immunosuppressant treatments including methotrexate and ciclosporin, in combination with best supportive care (BSC) (emollients and topical anti-inflammatory treatments), had been considered the standard of care for moderate-to-severe AD prior to the marketing authorisation of biological medicines in Ireland.

Dupilumab (Dupixent^®^) is a fully human immunoglobulin G4 monoclonal antibody that inhibits interleukin (IL)-4 and IL-13 signalling [2]. IL-4 and IL-13 are critical in the initiation and maintenance of the Th2 inflammatory pathway which plays a central role in the pathophysiology of AD [3]. Dupilumab was licensed for AD, by the European Medicines Agency (EMA), in 2017; the first biological medicinal product to be licensed for this indication. This approval was informed by the LIBERTY AD SOLO 1, LIBERTY AD SOLO 2 and LIBERTY AD CHRONOS clinical trial programmes [4–6]. A license was granted for use in moderate-to-severe AD in patients who are candidates for systemic therapy [2]. The subcutaneous injection is available in two strengths, a 200 mg formulation and a 300 mg formulation available as a pre-filled pen or pre-filled syringe [2].

At present, there are no guidelines for the treatment of AD in Ireland. The European Academy of Dermatology and Venereology (2018) recommends the use of dupilumab as a disease-modifying drug for patients with moderate-to-severe AD, in whom topical treatment is not sufficient and other systemic treatment is not advisable. Dupilumab should be combined with daily emollients and with topical anti-inflammatory treatments as needed [7].

### Dupilumab in the Irish healthcare setting

Reimbursement of dupilumab by the Health Service Executive (HSE) was previously sought by Sanofi Ireland (the applicant company) [8]. To inform the HSE reimbursement decision-making processes, the National Centre for Pharmacoeconomics (NCPE) completed three Health Technology Assessment (HTA) evaluations in different populations of patients with AD. The 2019 HTA assessed the cost-effectiveness of dupilumab for the treatment of moderate-to-severe AD in adult patients, who are candidates for systemic therapy [8]. The NCPE HTA considered two populations, i.e. the full-licensed population (herein the ‘full population’) and patients with refractory disease (herein the ‘refractory population’). The full population comprises adults with moderate-to-severe AD who are candidates for systemic therapy. The refractory population (a subgroup of the full population) comprises adults who are not adequately controlled by topical therapies and who are contraindicated to, intolerant of, have had an inadequate response to or for whom it is otherwise medically inadvisable to receive treatment with a systemic immunosuppressant. The NCPE considered methotrexate and ciclosporin to be appropriate comparators [8]. Incremental cost-effectiveness ratios (ICERs) varied depending on the population assessed; plausible ICERs ranged from €103,175 to €136,062 per quality-adjusted life year (QALY) in the full population and from €74,401 to €83,424 per QALY in the refractory population [8]. The NCPE estimated that the total annual cost of dupilumab to the HSE would be €19,911 per patient per year (inclusive of rebates, fees, and VAT) based on a price to wholesaler (PTW) of €1,153.85 for 4 weeks of supply [8]. We note that in 2019, the PTW of 4 weeks of supply of methotrexate at the maximum dose of 25 mg once weekly was about €5.24 and the PTW of ciclosporin at a dose of 200 mg twice daily was approximately €95.77 [9]. The NCPE estimated that the 5-year cumulative gross budget impacts would be about €51.9 million in the full population and approximately €38.3 million in the refractory population. The NCPE recommended that dupilumab not be reimbursed, at the submitted price, for the treatment of AD in adult patients [8].

In 2021, the HSE led the confidential price negotiations with the applicant company; these negotiations were informed by the NCPE HTA evaluation. As a result, the applicant company offered a confidential discount on the original submitted price of dupilumab. The NCPE concluded that the cost effectiveness of dupilumab had been improved as a result of this price discount. The HSE subsequently approved reimbursement of dupilumab for the treatment of moderate-to-severe AD in the refractory population (as defined within the NCPE HTA evaluations) [10]. This approval extends to two cohorts; adult patients aged 18 years and older (herein the ‘adult cohort’) and adolescent patients aged 12 to 17 years old (herein the ‘adolescent cohort’). The approval was subject to a HSE Managed Access Protocol (MAP) being implemented that would limit approval of treatment to patients with refractory disease only. The MAP was an initiative aimed at approving treatment for those patients in which the medicine was expected to be the most effective and cost-effective (versus standard of care) and also as a cost containment measure. The HSE-Medicines Management Programme (MMP) was tasked with developing and implementing this MAP. As per standard processes, the HSE-Primary Care Reimbursement Service (PCRS) is responsible for the High Tech (HT) ordering and management system which provides for the supply, dispensing and reimbursement of HT treatments (high-cost medicines which are initiated in a hospital setting) such as dupilumab through community pharmacies [11].

The HSE-MMP is a multi-disciplinary National Clinical Programme comprising clinicians, pharmacists and data analysts [12]. Against the backdrop of concerns regarding the affordability of medicines, the HSE-MMP has become the steward of Health Technology Management (HTM) in Ireland. HTM has been defined as ‘measures put in place to enhance the safe, effective, and cost-effective use of medicines thereby controlling utilisation and expenditure’ [13]. The HTM approach that Ireland has adopted, for the reimbursement of dupilumab, is comparable to the approach taken in other jurisdictions such as Canada, England and Scotland [14–17].

The eligibility criteria for patient-specific approval of dupilumab through the HSE were informed by European clinical guidelines, clinical trial efficacy data and the NCPE HTA evaluations [1, 4, 5, 7, 8, 18]. These eligibility criteria are outlined in the *HSE-Managed Access Protocol for High Tech Medicines for the treatment of moderate-to-severe atopic dermatitis* and include that patients [19]:

- Are aged 12 years or older at the time of application
- Have moderate-to-severe AD confirmed by an Eczema Area Severity Index (EASI) score of ≥ 16 at the time of application
- Previous immunosuppressant treatment:
  - Has failed or
  - Is not tolerated or
  - Is contraindicated
- Are in receipt of BSC for AD

Applications for individual patient approval of treatment with dupilumab, through the HT arrangement, are only considered when submitted by a HSE-approved prescriber [19]. Consultant dermatologists, in Ireland, who have read and formally agree to the terms of the MAP are eligible to be approved prescribers. All applications are reviewed on a case-by-case basis by the HSE-MMP. Once a patient is deemed eligible, by the HSE-MMP, for treatment with dupilumab, the approved prescriber is authorised to issue a prescription for the patient which can be dispensed by a community pharmacist through the HT arrangement.

Prior to the HSE reimbursement of dupilumab, the marketing authorisation holder (MAH) initiated an ‘early access programme’ (EAP). EAPs are operated at the discretion of the MAH. Here, the clinician would seek approval, from the MAH, for access to the drug for an individual patient. The MAH would approve access for that individual only if certain eligibility criteria (pre-defined by the MAH) are met. Applications to the HSE-MMP for individual supply of dupilumab for patients who are already on dupilumab, via the EAP, are processed according to the same HSE-MMP eligibility criteria applicable to all other patients.

### Database descriptions

There were two sources of data pertaining to the same cohort of patients included in this analysis.

Firstly, analysis was carried out on the data provided by consultant dermatologists in application forms submitted for the individual supply of dupilumab through the HT arrangement from 01 April 2021 to 31 March 2022 inclusive. This time frame represents the first year that dupilumab was available to patients in Ireland through the HT arrangement. The data analysed were anonymised and included information about the patient, their condition and previous treatments. Specifically, information captured and utilised included patient sex, patient date of birth, the duration since the patient diagnosis of AD, the EASI score and Children’s Dermatology Quality Life Index (CDLQI)/Dermatology Quality Life Index (DLQI) at the time of application, systemic immunosuppressant treatments previously received by the patient, the rationale for cessation of previous systemic immunosuppressant treatment and information regarding any contraindications the patient had for immunosuppressant treatment.

Furthermore, an evaluation of pharmacy claims data for dispensed items from the HSE-PCRS claims databases during the same time period as specified above was conducted. The HSE-PCRS claims databases comprise numerous databases. These include the General Medical Services (GMS) Scheme and Drugs Payment Scheme (DPS) databases which are of relevance here. Dupilumab is available to patients through the PCRS HT arrangement through their individual GMS or DPS eligibility. Patients with GMS scheme eligibility may access HT drugs for free at the point of care through this means-tested HSE community prescription drug scheme [11]. All other patients can access HT drugs through their DPS eligibility; here patients and their families have their monthly spend on approved prescribed drugs, medicines and medical and surgical appliances (including HT drugs) capped at a threshold value, currently €80 per calendar month [20]. Anonymised patient-level data, from both the GMS and DPS databases, were analysed from all claims submitted through the HT arrangement. The PCRS pharmacy claims database records details including age and sex of patient, and the cost, quantity, strength and form of drugs dispensed to patients eligible for the scheme.

### Aims of study

The aim of this study is to analyse the applications received during the first year of HSE reimbursement of dupilumab to determine:

- The percentage of patients (for whom an application to the HSE-MMP was made) who were eligible for HSE reimbursement and
- Certain characteristics (age, sex, time since first diagnosis and disease severity at the time of application) of this population.

## Methods

### Data analysis

Data from all application forms for individual reimbursement of dupilumab through the HT arrangement submitted to the HSE-MMP from 01 April 2021 to 31 March 2022 inclusive were identified. The sex and age distribution of the cohort were evaluated. The minimum and maximum ages, along with the mean age (± standard deviation), mean clinical scores (± standard deviation) and mean number of years between first diagnosis of AD and dupilumab initiation (± standard deviation) of the entire population and sub cohorts were established. Analyses were carried out using IBM SPSS Statistics^®^ version 27. Figures were generated using IBM SPSS Statistics^®^ version 27 and Microsoft Office Professional Plus 2013.

A retrospective analysis (within the above specified time period) of claims submitted through the HT arrangement for dupilumab was completed using the drug’s unique reimbursement code. These data were subsequently extracted from the HSE-PCRS claims database. All patients who accessed dupilumab under the GMS or DPS were included. The total number of individuals who had received at least one supply for dupilumab over the study period was determined.

As the data used in this study was anonymised, ethical approval was not required.

## Results

**Fig. 1:**
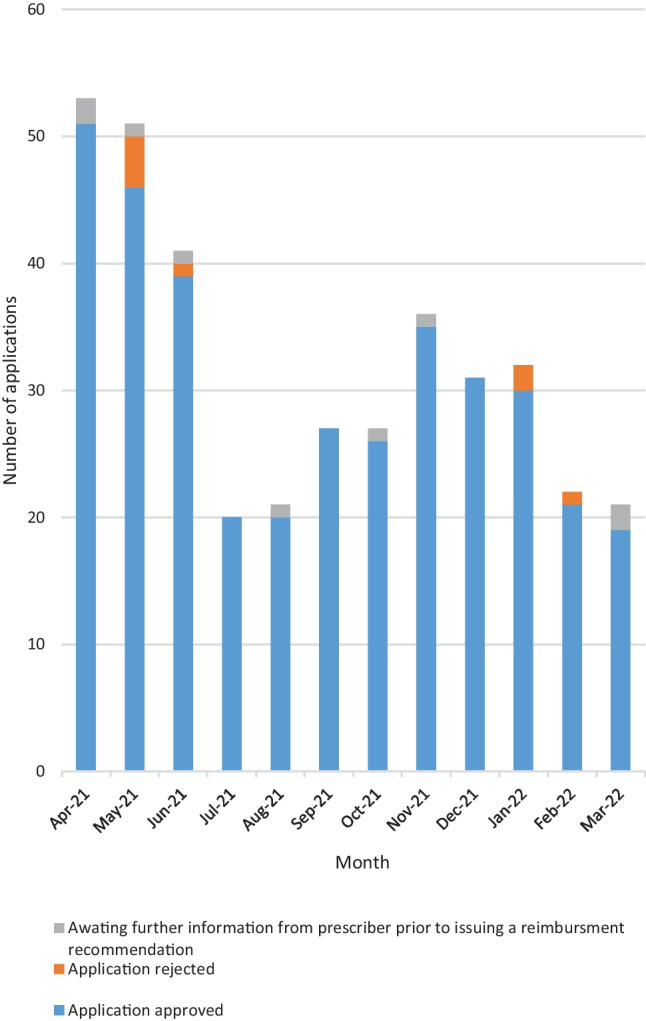
Monthly representation of outcomes of dupilumab reimbursement applications submitted to the Medicines Management Programme for review during the first year of reimbursement

From 01 April 2021 to 31 March 2022 inclusive, 382 applications for individual reimbursement of dupilumab through the HT arrangement were submitted for HSE-MMP review; 96% of patients (*n* = 365) were deemed eligible for treatment. Some 2% (*n* = 8) were rejected; HSE MAP eligibility criteria were not met. The HSE-MMP were awaiting clarifications on uncompleted application forms in 2% (*n* = 9) of applications at the time of analysis (01 November 2022). Figure 1 demonstrates the monthly breakdown of application outcomes.

Of the 382 applications submitted, 88 (23%) highlighted that the patient was a participant in an EAP. Some 96% (*n* = 84) of these applications were approved; 3% (*n* = 3) were rejected given that eligibility criteria were not met. The HSE-MMP was awaiting clarification of information for the remaining application.

In total 65% (*n* = 238) of patients deemed eligible for treatment were male and 87% (*n* = 316) were adults. The mean age of all patients was 35 years (range 12 to 79 years); 65% were aged between 16 and 44 years. The mean number of years between diagnosis of AD and approval for treatment with dupilumab was 22.65 years, with a range of 1 to 78 years (Table 1).

**Table 1:** Approved patient characteristics

	**Full population** ***n* = 365**		**Adolescent cohort** ***n* = 49**		**Adult cohort** ***n* = 316**	
**Male *n* (%)**	238 (65.20%)		31 (13.03%)		207 (86.97%)	
**Female *n* (%)**	127 (34.80%)		18 (14.17%)		109 (85.83%)	
	**Mean**	**Standard deviation**	**Mean**	**Standard deviation**	**Mean**	**Standard deviation**
**Age of patient (years)**	34.88	15.35	15.12	1.72	37.95	14.2
**Number of years between first atopic dermatitis diagnosis and dupilumab initiation**	22.65	14.73	12.43	3.91	24.24	15.16
Baseline EASI score*	28.72	11.25	26.01	9.61	29.21	11.47
Baseline CDLQI/DLQI score*	19.14	6.38	15.95	5.89	19.72	6.31

*CDLQI* Children’s Dermatology Life Quality Index, *DLQI* Dermatology Life Quality Index, *EASI* Eczema Area Severity Index

^*^EASI and CDLQI/DLQI analysis on newly initiated patients only *n* = 281 (*n* = 43 in adolescent cohort, *n* = 238 in adult cohort)

**Fig. 2:**
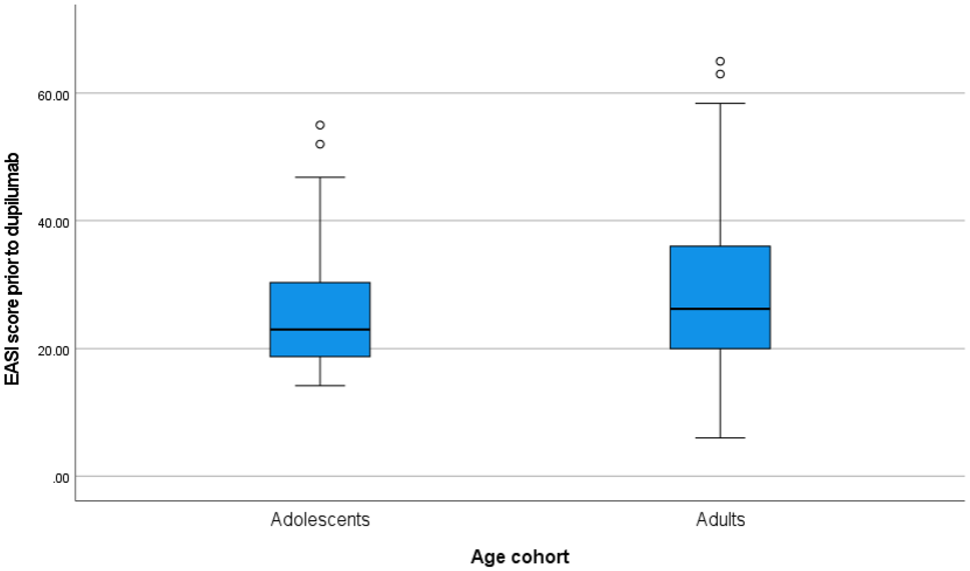
Baseline Eczema Severity Index Scores (EASI) of approved patients prior to initiating treatment with dupilumab

All patients who were deemed eligible for treatment were reported to have moderate-to-severe AD. Patients who participated in EAPs were excluded from the following analyses as their EASI and CDLQI/DLQI scores at the time of application reflected the severity of their AD whilst on treatment with dupilumab. Of the 281 patients who were eligible and who were dupilumab treatment naïve, the mean EASI score was 28.72 (Fig. 2). The EASI score determines severity based on physicians assessment of the redness, thickness, excoriation and lichenification of skin and the percentage of skin involvement in four areas (the head, trunk, arms and legs). The total score will fall between 0 and 72 with the numerical value classifying the severity of the disease; a score of 7 or lower indicates mild disease, a score of 8 to 21 indicates moderate disease, a score of 22 to 50 indicates severe disease and a score of 56 to 72 indicates very severe disease [21].

**Fig. 3:**
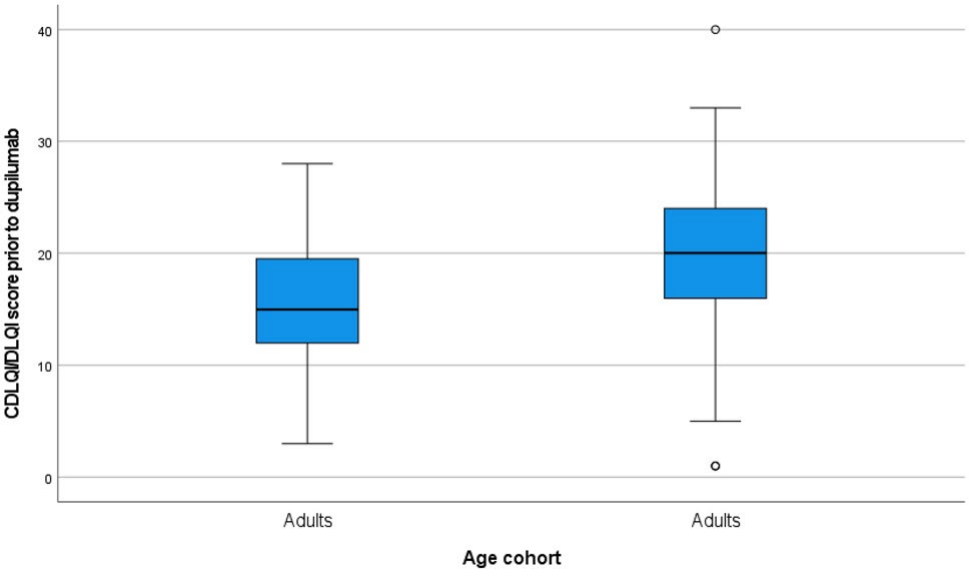
Baseline Children’s Dermatology Quality of Life Index Score (CDLQI)/Dermatology Quality of Life Index Score (DLQI) of approved patients prior to initiating treatment with dupilumab

The mean CDLQI/DLQI score was 19.72 (Fig. 3). The CDLQI/DLQI is a validated, dermatology-specific quality-of-life questionnaire consisting of ten questions concerning patients’ perceptions of the impact of skin diseases on different aspects of their health-related quality of life over the last week. The scores may be interpreted as follows: no effect on life if score is between 0 and 1, small effect if score is between 2 and 6, moderate effect if score between 7 and 12, very large effect if score is between 13 and 18 and extremely large effect if score is between 19 and 30 [22, 23].

Patients deemed eligible for treatment who had previously received but had not responded to standard-of-care systemic immunosuppressant treatment for AD had trialled up to five different medicines (mean = 1.6). Such treatments included methotrexate (*n* = 252), ciclosporin (*n* = 147), azathioprine (*n* = 82), mycophenolate mofetil (*n* = 58), Janus kinase inhibitors (*n* = 28), ustekinumab (*n* = 4), apremilast (*n* = 1) and thalidomide (*n* = 1). The majority of patients who were eligible for treatment (*n* = 294; 81%) had an inadequate response to at least one immunosuppressant treatment. An intolerance to at least one immunosuppressant treatment was reported in 92 patients (25%) whilst a contraindication to immunosuppressant treatment was reported in 46 patients (13%). Some patients were reported to have had combinations of inadequate response, intolerance and contraindication to immunosuppressant treatment.

**Fig. 4:**
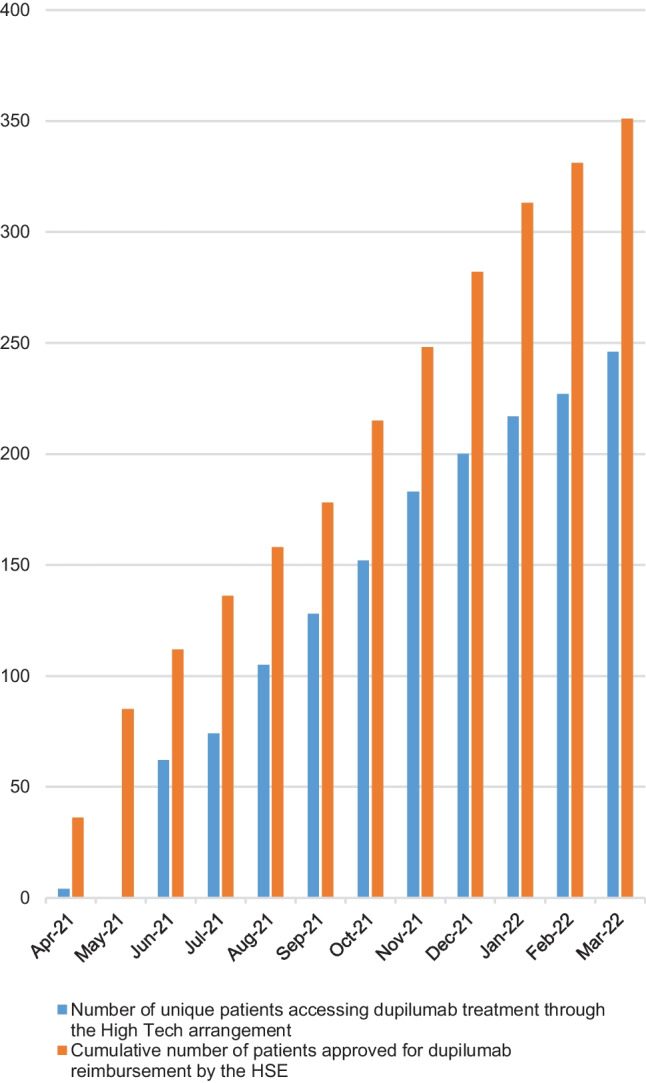
Cumulative number of patients approved for reimbursement of dupilumab and the cumulative number of patients accessing treatment through the High Tech arrangement

Over the 12-month period analysed, there were 1780 requests for dupilumab submitted by community pharmacies for supply to patients who were deemed eligible through the HT arrangement. These requests were placed on behalf of 305 individual patients. Total expenditure based on PTW during the first year of reimbursement amounted to €2,439,640 [24]. Figure 4 demonstrates the cumulative number of patients deemed eligible for dupilumab on the last day of each month compared to the number of patients who had a request for a supply of dupilumab placed on their behalf by a community pharmacy at any stage throughout that month.

## Discussion

The NCPE’s HTA concluded that the ICER for dupilumab in adult patients with moderate-to-severe, refractory AD exceeded €66,000 per QALY. This is well above the threshold of €45,000/QALY that has been used within the decision making processes in Ireland [8, 25]. The predicted gross budget impact estimate was €38.3 million over 5 years, with the licence extension granted to the adolescent population expected to increase the budget impact substantially. In view of the failure to demonstrate cost-effectiveness at the proposed PTW and the large estimated budget impact, the HSE reimbursed dupilumab subject to the implementation of a MAP. This managed access approach has proven successful, in Ireland, for other interventions including the restriction of reimbursement of lidocaine patches for their licensed use only which has ensured evidence-based prescribing and reduced the expenditure from a peak of over €27 million per annum to just over €2 million per annum [26].

Patient access to novel treatments is another important consideration for reimbursement; 96% of applications submitted by dermatologists were approved for reimbursement of dupilumab by the MMP. These patients ranged in age from the minimum approvable age of 12 years up to 79 years. Although licensed and reimbursed for moderate-to-severe AD, the results indicate that patients deemed eligible for treatment here primarily experienced severe AD, having a mean EASI score of 28.72 [27]. Severe AD has been documented as a difficult-to-treat condition in which the available immunosuppressant treatments are associated with significant potential toxicities, drug interactions and contraindications and the utilisation of BSC alone is typically ineffective [28]. Patients, who were deemed eligible for treatment with dupilumab, had previously been treated with up to five systemic treatments for their AD, many off-label, prior to approval for supply of dupilumab. A mean of 22.65 years had elapsed between approved patient original diagnoses of AD and their approval for this treatment, highlighting the absence of novel effective therapies in this therapeutic area. An interventional study into the long-term safety and sustained efficacy of dupilumab in adults over the course of 4 years has shown favourable results which support the role of dupilumab as continuous long-term treatment for patients with moderate-to-severe AD [29]. Preliminary results for the adolescent population show similar outcomes [18].

European guidelines state that ciclosporin is usually considered as the first-line option for patients requiring immunosuppressant treatment as it is licensed for the treatment of AD. However, based on the information submitted by consultant dermatologists in the application forms, methotrexate was the most frequently used immunosuppressant treatment in this cohort [7, 8]. The results show that almost 70% of eligible patients had previously received treatment with methotrexate; about 40% had previously received treatment with ciclosporin. To our knowledge, there are no published head-to-head studies between dupilumab and methotrexate or ciclosporin for the treatment of AD. Seger et al. completed a systematic literature review on the relative efficacy of systemic treatments for AD which concluded that ciclosporin and dupilumab are both effective in improving clinical severity of AD [30]. Drucker et al. conducted a network meta-analysis which had a similar conclusion; dupilumab appeared to have similar efficacy to ciclosporin and both were superior in improving clinical severity of AD when compared to methotrexate and azathioprine [31]. The HSE’s MAP outlines that, if not intolerant to or contraindicated for immunosuppressant treatment (azathioprine, ciclosporin, methotrexate, mycophenolate mofetil), at least one previous immunosuppressant treatment must have been ineffective prior to HSE-MMP authorisation of patient specific approval for supply of dupilumab [19]. This reimbursement criterion is comparable to guidance from the National Institute for Health and Care Excellence (NICE) in England and Scottish Medicines Consortium (SMC) in Scotland [15, 16]. Of interest, dissimilar to the MAP criteria, Canada’s Drug and Health Technology Agency (CADTH) guidance states that both methotrexate and ciclosporin must be deemed ineffective prior to approval dupilumab for a specific patient. In our study, just 25% of patients, deemed eligible by the HSE-MMP, have been reported to have failed to respond to previous immunosuppressant treatment with both methotrexate and ciclosporin therapy [14]. Overall, just 2% of patients did not meet the eligibility criteria and thus were not eligible for treatment; these applications did not demonstrate that the patients had moderate-to-severe AD refractory to immunosuppressant treatment or that such treatment was unsuccessful, contraindicated or unsuitable due to patient intolerance.

It is seen that 365 patients were approved for treatment with dupilumab in the first year, almost a quarter of whom were accessing the treatment through an EAP prior to the implementation of the MAP. These EAPs are established and operated at the discretion of pharmaceutical companies; the HSE is not involved in the establishment of EAPs. In a report considering patient access to novel medicines in the European environment, it is suggested that pharmaceutical companies who operate EAPs should accept risks such as continuation of the programme if reimbursement of the intervention is declined [32]. We noted that HSE views are consistent with this based on a memorandum from May 2018 which highlights the necessity to ensure that appropriate arrangements are agreed and in place for the aftercare of patients post access programmes and that such arrangements are clearly established and transparent to all parties (including patients) in advance. In this particular case, the MAH committed to continued access to dupilumab for patients on the EAP until the HSE made a recommendation for individual patients and also in the event of a negative recommendation for an individual patient. However, it is the experience of the MMP that this is not the approach taken by all MAHs. Thus, in reality, it is possible that future patients who have availed of certain treatments via EAPs may not fulfil the MAP criteria for approval of supply.

Interestingly, Fig. 4 demonstrates that not all patients who are deemed eligible for treatment with dupilumab treatment access it straightaway. In March 2022, only 70% of approved patients had an order for dupilumab placed on their behalf by a community pharmacy. It is not clear, at this time, if this represents a delay in starting treatment or a decision (made by the prescriber or patient) not to start treatment at all. Further, it is not clear why either of these pathways would be followed. Also, it is useful to note, that, currently it is understood that adverse events associated with dupilumab tend to be mild, self-limiting and manageable [4, 8]. Of interest, a study which covered 13 European countries (including Ireland) demonstrated there is a positive link between an individuals increased out-of-pocket payment and the probability that individuals will forego or delay treatment [33]. Whilst access to dupilumab for approved patients through the means-tested GMS is free at the point of care, those without such eligibility are subject to the €80 per month co-payment through the DPS. Over half, 60% (*n* = 218), of approved applications related to patients without GMS eligibility. The monthly co-payment is mandatory for all DPS patients, including those who had previously accessed treatment free at the point of care through an EAP. The effect of out-of-pocket expense on adherence with dupilumab therapy is an area that warrants further investigation.

In total, 96% of patients were deemed eligible for treatment; 4% were deemed ineligible for treatment. In terms of cost containment and evidence-based prescribing, it seems reasonable to assume that this 4% represents an underestimation of the number of patients for which treatment would have been initiated if the HSE-MMP MAP had not been implemented. Any instances, where a prescriber considered initiation of treatment, but reconsidered this on review of the MAP eligibility criteria, have not been captured here. This is an added utility of the MAP process that we cannot measure.

A strength of this study is the availability of robust pharmacy claims data on all patients accessing treatment through the HT arrangement in Ireland. As dupilumab has only been available through the HT arrangement in Ireland since April 2021, further research will be required to determine the long-term utilisation and expenditure on this treatment.

There was a significant ransomware attack on some HSE systems in May 2021; some approved prescribers may have had limited access to electronic systems for a time period following the attack and it is unknown if this had an influence on the number of applications submitted around that time, making this is a potential limitation of the study.

## Conclusion

As it was determined that dupilumab was not cost-effective for the treatment of moderate-to-severe AD at the proposed PTW, subsequent confidential price negotiations and a MAP were required to facilitate the reimbursement of this high-cost medicine. Over the first year of reimbursement, 96% of individual patient applications for dupilumab were deemed eligible by the HSE-MMP. The majority of these patients experienced severe AD and had previously received but had not responded to standard-of-care immunosuppressant treatments; thus the MAP achieves its aim of containing cost whilst facilitating access for those patients in whom it is anticipated the medicine will be effective.

It was noted that the number of patients deemed eligible for treatment is higher than the number of patients who access the treatment. The reason for this is unknown and requires further investigation. Outcome data in relation to approved patients will be gathered and will provide insight into the efficacy of dupilumab in this real-world cohort of patients accessing treatment through the HSE MAP.

## Data Availability

The data that support the findings of this study are available from the Health Services Executive’s Primary Care Reimbursement Service (HSE-PCRS), but restrictions apply to the availability of these data, and so are not publicly available.
